# Une lésion labiale traumatique insolite

**DOI:** 10.11604/pamj.2023.45.52.39776

**Published:** 2023-05-23

**Authors:** Romaric Beheton, Arsène Coulibaly

**Affiliations:** 1 Clinique Universitaire de Chirurgie Maxillo-faciale et d’Odonto-stomatologie, Centre National Hospitalier et Universitaire Hubert Koutoukou Maga (CNHU-HKM) de Cotonou, Cotonou, Bénin,; 2 Centre Hospitalier Universitaire Régional de Ouahigouya, Ouahigouya, Burkina Faso

**Keywords:** Traumatisme, incarcération, lèvre supérieure, Trauma, incarceration, upper lip

## Abstract

Trauma to the lips is the most common type of facial injury. The prominence of the lips and the close correlation with the teeth, make it a vulnerable aesthetic unit. We here report the case of a 20-year-old student presenting two hours after maxillofacial trauma following a traffic accident involving a head-on collision between two motorcyclists. The student had fallen on the handlebars of his motorbike injuring his upper lip. Maxillofacial examination showed involvement of the nasogenial sulcus with elevation of the right nasal wing, a notch in the right upper hemi-lip, well colored, and whose endo-buccal extension was embedded in the vestibule, giving an appearance of “right unilateral cleft lip”. Radiological evaluation showed air-fluid levels with solution of continuity of the medial wall of the right maxillary sinus. The diagnosis of traumatic incarceration of the right upper hemi-lip with no displaced fracture of the right maxilla was retained. This can cause unilateral or incomplete right upper cleft lip, or a wound with loss of substance involving the right upper hemi-lip. It is a very uncommon lesion of the upper lip requiring urgent repair, given the risk of ischemia and labial necrosis. The patient was operated under locoregional anesthesia. The right upper hemi-lip was embedded with incarceration due to non-displaced fracture at the level of the nasal notch. Disincarceration was performed, resulting in minimal labial loss with a vestibular detachment, repaired by direct suture. The patient’s outcome was good.

## Image en médecine

Les traumatismes des lèvres occupent une place prépondérante des plaies faciales. La proéminence des lèvres et le rapport étroit contracté avec les dents, en font une unité esthétique vulnérable. Un élève de 20 ans a été reçu 02 heures après un traumatisme maxillo-facial suite à un accident de la circulation par collision frontale entre deux motocyclistes, avec chute et réception de la lèvre supérieure sur le guidon de sa moto. L´examen maxillo-facial notait un comblement du sillon naso-génien avec surélévation de l´aile nasale droite, une encoche de l´hémi-lèvre supérieure droite, bien colorée, et dont le prolongement endobuccal était enfoui dans le vestibule, donnant un aspect de « fente labiale unilatérale droite ». Le bilan radiologique a noté un niveau hydro-aérique avec une solution de continuité de la paroi médiale du sinus maxillaire droit. Le diagnostic d´incarcération traumatique de l´hémi-lèvre supérieure droite avec fracture du maxillaire droit non déplacée est retenu. Ceci peut se discuter avec une fente labiale supérieure droite unilatérale ou incomplète, ou une plaie avec perte de substance de l´hémi-lèvre supérieure droite. C´est une lésion très inhabituelle de la lèvre supérieure nécessitant une réparation en urgence vue le risque d´ischémie et de nécrose labiale. Sous anesthésie locorégionale, l´exploration notait l´hémi-lèvre supérieure droite enfouie et incarcérée à travers une fracture non déplacée située à hauteur de l´incisure nasale. La désincarcération est réalisée, occasionnant une perte de substance labiale minime avec un décollement vestibulaire, réparés par suture directe. L´évolution était bonne.

**Figure 1:**
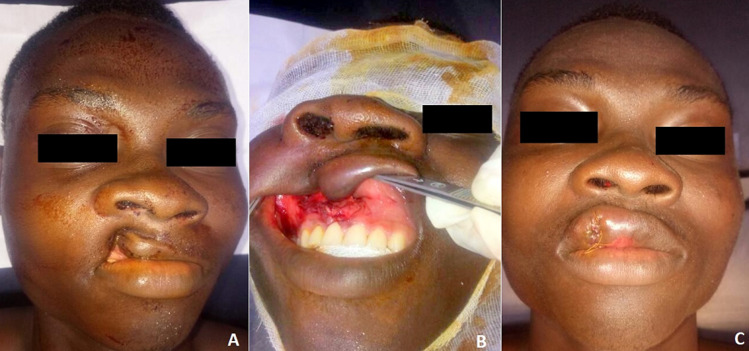
A) fente labiale unilatérale; B) incarcération de l'hémi-lèvre supérieure; C) aspect après réparation

